# Supplementary material for: Countering the potential re-emergence of a deadly infectious disease—Information warfare, identifying strategic threats, launching countermeasures
Source: PLoS One. 2021 Aug 20;16(8):e0256014. doi: 10.1371/journal.pone.0256014 (PMC8378755; doi:10.1371/journal.pone.0256014)
Supplement: S1 Text — The proof proceeds in the following sequence. (1) We provide a Continuous Time Markov Chain (CTMC) formulation for the stochastic version of our system. (2) We present a classical result of probability theory that guarantees convergence of a CTMC to a system of differential equations provided the CTMC satisfies some regularity conditions. (3) and (4) We prove that the CTMC for our system satisfies the regularity conditions. (5) We show that the system of differential equations in the classical result is the CEDE for our system. (PDF) [file pone.0256014.s001.pdf]

C Proof of convergence of the stochastic state distribution to CEDE solution

We now consider stochastic evolutions for the joint spread of disease and opinion in presence of four different combinations of countermeasures: 1) no countermeasure 2) drug only 3) vaccine only 4) both drug and vaccine. We model the stochastic evolutions as a Continuous-Time Markov chain (CTMC) (Sec C.1), CTMCs have been extensively used in modeling the spread of infectious diseases (e.g., Chapter 2, p. 28, [25]). We subsequently present a classical result from probability theory that shows that for a class of CTMC the fraction of individuals in different states converge to the solution of a set of differential equations. The same result also shows how to construct those differential equations given the transition rates of such a CTMC (Sec C.2). We next show that the CTMC representing the system we consider belongs to that class (Sec C.3, C.4). Finally, we obtain the differential equations from the classical result for our system, which turn out to be the CEDE as described in Sec A (Sec C.5).

C.1 A Continuous Time Markov Chain formulation

We consider that there are v alive individuals in the system at the start of the period we are interested in. Recall that an individual is characterized by the cluster he inhabits, his cooperativity, stage of the disease, preemption, immunocompetency or immunodeficiency 10). The state of the system is a vector specifying the number of individuals corresponding to each possible combination of the attributes described above. Let the number of combinations be k. Then the state vector belongs in $E_v \subset \mathbb{Z}^k$. The state of the system changes whenever an individual changes one of his characterizing attributes. We assume that once an individual is infected, he transitions through successive stages of the disease until he is preempted or recovered or dies; duration in each stage is an exponentially distributed random variable. The stage of the disease does not change once an individual is preempted, recovers, or dies. Similarly, all the following durations are also exponentially distributed random variables: 1) times that each individual spends in a cluster before moving to another; 2) time before he is preempted (that is, receives vaccine or drug which is effective in him, that is, cures him if he already has the disease and/or prevents him from developing the disease in future); 3) times between successive physical interaction or opinion spread between a given pair of individuals in the same or neighboring clusters (i.e., clusters g, h such that $\phi_{gh} > 0$, $\alpha_{gh} > 0$). If all these exponentially distributed random variables are independent, the sojourn time of the system in each state is also exponentially distributed. Such a system constitutes a CTMC, parameterized by v.

C.2 A classical result from the theory of one parameter Markov chains

We consider a family of Markov chains, $X_v(t)$, parameterized by a positive number v, with state space $E_v \subset \mathbb{Z}^{\mathbf{k}}$, where $\mathbb{Z}^{\mathbf{k}}$, is the set of k-dimensional vectors with integer components. If for example v is

the number of individuals in the system, $X_v(t)/v$ is the vector representing the fraction of individuals in various system states. Let $q_{m,m+l}$ be the infinitesimal parameters (transition rates) between states $m, m+l$ for this Markov chain. Here, $l, m \in \mathbb{Z}^{\mathbf{k}}$, l is the vector deviation between the current state m and the next state $l+m$. Also, $|l|$ captures the magnitude of this deviation. We refer to l as the *transition vector*. Let there exist continuous functions $f(x,l)$, $x \in \mathbb{R}^{\mathbf{k}}$, such that:

$$q_{m,m+l} = vf\left(\frac{1}{v}m, l\right), \qquad l \neq 0. \tag{34}$$

We denote $f(\cdot)$ as the *transition rate functions* given the above relation with the transition rates. Note that $x = m/v$, thus x is a k-dimensional vector with components in $\mathbb{R}^{\mathbf{k}}$. It may be envisioned as a vector representing the fraction of individuals in various system states. Define

$$F(x) = \sum_l l f(x,l). \tag{35}$$

The following Theorem is a classical result of probability theory, stated and proved as Theorem 3.1 of [30].

Theorem: Suppose there exists an open set $E \subset \mathbb{R}^{\mathbf{k}}$ and a constant M_E such that

$$|F(x) - F(y)| < M_E|x-y| \qquad x, y \in E, \tag{36}$$

$$\sup_{x\in E} \sum_l |l| f(x,l) < \infty, \tag{37}$$

$$\lim_{d\to\infty} \sup_{x\in E} \sum_{|l|>d} |l| f(x,l) = 0. \tag{38}$$

Then for every trajectory $X(s, x_0)$ satisfying

$$X(0, x_0) = x_0, \tag{39}$$

$$X(s, x_0) \in E \qquad 0 \le s \le t, \tag{40}$$

$$\frac{\partial}{\partial s} X(s, x_0) = F(X(s, x_0)), \tag{41}$$

$\lim_{v\to\infty} v^{-1} X_v = x_0$ implies for every $\delta > 0$,

$$\lim_{v\to\infty} P\left\{\sup_{s\le t}\left|\frac{1}{v}X_v(s) - X(s, x_0)\right| > \delta\right\} = 0. \tag{42}$$

We now explain the significance of the above classical result.

First note that (36) - (38) constitute *regularity conditions*. Condition (36) requires that $F(\cdot)$ is

Lipschitz-continuous, informally, this means that it changes slowly with change in the argument. Intuitively, this condition follows only if $f(\cdot)$ are also slowly varying functions, since $F(\cdot)$ is obtained from the transition rate functions $f(\cdot)$, via (35). Condition (37) essentially requires that the transition rates be finite. Condition (38) will hold if transitions do not happen to "far off" states, that is, when there is a large deviation $|l|$ between two states, the system does not directly move from one to the other - thus, the state transitions are gradual.

Now note that $X(s, x_0)$ is a k-dimensional vector in $E \subset \mathbb{R}^{\mathbf{k}}$. Its derivative with respect to time s in left hand side (LHS) of (41) is a k-dimensional vector of time derivatives of its k components. Since $l \in \mathbb{Z}^k$ and $f(\cdot)$ is a scalar function in $\mathbb{R}$, from (35), right hand side (RHS) of (41) is a vector in $\mathbb{R}^k$. Thus (41) is a system of k differential equations. The initial conditions of these differential equations are given by (39).

The theorem states that if a Markov chain satisfies the regularity conditions (36) - (38), at each time s, the fractions of individuals in various states given by the stochastically evolving vector of fraction of individuals in different states, $X_v(s)/v$, converges to the solution of the differential equations (41) as number of individuals v approaches infinity (refer to (42) for the specific convergence guarantees). And the differential equations (41) may be obtained from the transition rate functions via (35).

Thus, we seek to show that the CTMC we consider satisfies the regularity conditions (36) - (38), and the transition rates of the CTMC provide us the CEDE following (34) and (35).

C.3 Obtaining the transition rate functions $f(\cdot)$

We classify state transitions into 5 broad categories: those that (1) spread infection (convert susceptibles to early incubators) (2) spread opinion (convert non-cooperatives to cooperatives) (3) change location (mobility from one cluster to another) (4) change stage of disease (natural progression of disease) (5) preempt (vaccinate or start drug treatment and those are effective). We characterize the transition rate functions for the transitions in each category, using (34).

First, in each transition a particular component of the state vector increases by 1 and another decreases by 1 - the corresponding components of the transition vector l are $1, -1$ respectively and the rest of the components are 0. We refer to all such vectors in $\mathbb{Z}^{\mathbf{k}}$ as *valid transition vectors.* Thus $q_{m,m+l} = 0$ if l is not a valid transition vector, e.g., if $|l| > \sqrt{2}$ considering an Euclidean (L^2) norm for $|l|$. Thus

$$f(x, l) = 0 \quad \text{if } l \text{ is not a valid transition vector, e.g., if} \quad |l| > \sqrt{2}. \tag{43}$$

We now consider the valid transition vectors l and obtain the $f(\cdot)$ for the 5 categories of transitions enumerated above.

Spread infection. Consider the transition of a susceptible in cluster g to an early incubator of the same type (cooperative/non-cooperative, immunocompromised/immunocompetent, same cluster). The transition rate $q_{m,m+l}$ is $m_{sg} \sum_{h:\tilde{\phi}_{hg}>0} \phi^{(v)}_{hg} m_{ih}$ where m_{sg} are the number of susceptibles of the type in question in cluster g, m_{ih} ("i" stands for infections) are the number of infectious individuals, i.e., those in prodrome, early and late rash stages, of all types, in cluster h, $\{\tilde{\phi}^{(v)}_{hg}\}$ are the disease spread rates from cluster h to g in systems with v individuals. Here, $\{\tilde{\phi}^{(v)}_{hg}\}$ is the rate of the exponential random variable that represents the time between successive infection spreading contacts between a pair of individuals, a susceptible in cluster g and an infectious individual in cluster h. More specifically, $\{\tilde{\phi}^{(v)}_{hg}\}$ represents the rate of physical contact between individuals in the pair described as above times the probability that such a contact infects the susceptible. If a system has a large number of individuals, typically, contacts between each given pair becomes less frequent (e.g., in a small community all individuals know each other, but in a large community like an urban region very few individuals know each other). Thus, the contact rates are indexed by v, in fact, we will assume that $\tilde{\phi}^{(v)}_{hg} = \phi_{hg}/v$ where ϕ_{hg} is a constant independent of v. Note that $\{\phi_{hg}\}$ are the disease spread rates used in the CEDE. Since $q_{m,m+l} = vf(\frac{m}{v}, l)$, following (34), we have $f\left(\frac{m}{v}, l\right) = \frac{m_{sg}}{v} \sum_{h:\phi^{(v)}_{hg}>0} \phi_{hg} \frac{m_{ih}}{v}$. Thus, since $x = \frac{m}{v}, x_{sg} = \frac{m_{sg}}{v}, x_{ih} = \frac{m_{ih}}{v}$, $f(x, l) = x_{sg} \sum_{h:\phi_{hg}>0} \phi_{hg} x_{ig}$.

Spread opinion. Consider the conversion of a non-cooperative in cluster g to a cooperative of the same type (disease stage, immunocompromised, cluster etc.) due to exchange of opinions with a cooperative of any type in the same or neighboring cluster h (i.e., if the opinion spread rate $\alpha^{(v)}_{hg} > 0$). Proceeding similarly to the previous case, $f(x, l) = x_{bg} \sum_{\alpha_{hg}>0} \alpha_{hg} x_{ah}$, where x_{bg} represents the component of x that corresponds to the non-cooperative of the type in question and in cluster g, x_{ah} represents the sum of the components corresponding to cooperatives of all types in cluster h, and $\tilde{\alpha}^{(v)}_{hg} = \alpha_{hg}/v$ where, like for ϕ_{hg}, we assume that α_{hg} does not change with v. Note that $\{\alpha_{hg}\}$ are the opinion spread rates used in the CEDE.

Change location. Consider the transition of an individual from a cluster g to another cluster h. This happens because of mobility. Let $\kappa^{(v)}_{gh}$ be the mobility rate of individuals from cluster g to h i.e the rate of exponential distribution representing the sojourn time of an individual in cluster g before he moves to cluster h, when there are v individuals in the system. Note that mobility rate of individuals depend on their travel requirements which do not usually depend on the total number of individuals in the region. We therefore assume that $\kappa^{(v)}_{gh}$ does not change with v, and denote it by κ_{gh}. Note that $\{\kappa_{gh}\}$ are the mobility rates used in the CEDE. Thus, $q_{m,m+l} = \kappa_{gh} m_g$ where m_g is the number of individuals in cluster g of the type in question. Thus, $f\left(\frac{m}{v}, l\right) = \kappa_{gh} \frac{m_g}{v}$. Thus, since $x = \frac{m}{v}$, $f(x, l) = \kappa_{gh} x_g$, following (34) where x_g is the component of x of the type in question in cluster g.

Disease progression. Consider the progression of an individual from one stage of the disease to another. Following similar arguments as before $f(x,l) = \zeta x_g$ where x_g represents the component of x corresponding to the type (stage of the disease, cooperativity, cluster, immunocompromise) of the individual in question, ζ is the rate of the exponential random variable representing the sojourn time in the stage of the disease in question, clearly it does not depend on v. Considering our case, ζ would be ω, β, γ, ρ, and δ (Table 6) for early incubation, late incubation, prodrome, early and late rash stages respectively.

Preemption. Consider the preemption of individuals. Finally, proceeding similarly, one can show that the transition rate function for preemption is $f(x,g) = \tau x_g$ where x_g is the component of x representing the type (disease, cooperativity, cluster, immunocompromise) of the individual in question. Note that the preemption rate τ will depend on the combination of countermeasures and the stage of the disease (the latter is part of the type of the individual).

A compact representation for the transition rate function $f(\cdot)$: Considering all the possible transitions, we have shown that $f(\cdot,l)$ is quadratic function for some l, and linear for others. A compact representation would be:

$$f(x,l) = \sum_{g,h} \gamma^{l,g,h} x_g x_h + \sum_h r^{l,h} x_h \tag{44}$$

Here, $\gamma^{l,g,h} = 0$, for those transitions (i.e., l) for which $f(\cdot,l)$s are linear, $r^{l,h} = 0$ for those transitions (i.e., l) for which $f(\cdot,l)$s are quadratic. Also, note that both the quadratic and linear $f(\cdot,l)$ involve only some terms, the coefficients $\gamma^{l,g,h}, r^{l,h}$ would be 0 for the rest. Note that (43) is a special case of (44) - $\gamma^{l,g,h}, r^{l,h}$ would be 0 if $|l| > \sqrt{2}$ (more generally, if l is not a valid transition vector).

C.4 The transition rate functions $f(\cdot)$ for our system satisfies the regularity conditions (36) - (38)

Since each component of the system state vector m is a certain number of individuals and v is the total number of individuals in the system, the components of m/v are in $[0,1]$. Accordingly the argument x of $f(\cdot)$ and $F(\cdot)$ has components in $[0,1]$ via (34) and (35). Thus, we consider E to be the set of vectors in $\mathbb{R}^{\mathbf{k}}$ whose components are in $[0,1]$.

We now show that the condition (36) holds in our case, that is, $F(\cdot)$ is a Lipschitz continuous function. Now, from (35) and (44), $F(\cdot)$ is a linear combination of a finite number of quadratic and linear functions. It follows from standard real analysis that 1) both quadratic and linear functions of vectors in E are Lipschitz continuous (since components of the vectors in E are in $[0,1]$) and 2) linear combinations of

a finite number of Lipschitz continuous functions is Lipschitz continuous. Thus condition (36) follows. Next, we provide the proofs of the above standard results for completeness.

First consider that a function $f_1(x) = x_i$. Then $|f_1(x) - f_1(y)| = |x_i - y_i| \leq |x - y|$. The last step follows from the property of vector norms. Thus, Lipschitz continuity of linear functions follow. Now consider that a function $f_1(x) = x_i x_j$. Then $|f_1(x) - f_1(y)| = |x_i x_j - y_i y_j| = |x_i x_j - x_i y_j + x_i y_j - y_i y_j| = |x_i(x_j - y_j) + (x_i - y_i) y_j| \leq |x_i||x_j - y_j| + |y_j||x_i - y_i|$. The last step follows from triangle inequality of norms (e.g., Chapter T, p. 383 - 384, [43]). Thus, $|f_1(x) - f_1(y)| \leq 2|x - y|$, since $|x_i| \leq 1, |y_j| \leq 1,$ as $x, y \in E$. Thus, Lipschitz continuity of quadratic functions follow. We now argue that linear combinations of a finite number of Lipschitz continuous functions is Lipschitz continuous. Let $\bar{f}(x) = \sum_i a_i f_i(x)$, where $f_1(\cdot), f_2(\cdot), \ldots$ are all Lipschitz continuous. Thus, $\bar{f}(x) - \bar{f}(y)$ $= \sum_i a_i(f_i(x) - f_i(y))$. Thus, $|\bar{f}(x) - \bar{f}(y)| \leq (\sum_i |a_i| b_i)|x - y|$. The last step follows from the triangle-inequality and Lipschitz-continuity of $f_1(\cdot), f_2(\cdot), \ldots$. The last step establishes the Lipschitz continuity of $\bar{f}(x)$.

From (43), and since there are only a finite number of valid transition vectors, $f(\cdot, l)$ is non-zero for only a finite number of vectors l. Thus, (37) follows from (44) and since $0 \leq x_i \leq 1$ for each component i and $x \in E$. Finally, (38) follows directly from (43).

C.5 Deriving the CEDE

We now only need to show that (41) constitute the CEDE for various combinations of the countermeasures deployed.

We had argued in Section C.2 that (41) is a system of k differential equations. Note that the CEDE also has k differential equations where k is the number of system states.

Recall that in each valid transition vector l all but 2 components are non-zero, and one of these non-zero components is 1 and another is -1. Thus, the RHS of the p-th of these differential equations, that is the pth component of $F(X(s, x_0), l)$ is:

$$\sum_{l:l_p=1} f(X(s, x_0), l) - \sum_{l:l_p=-1} f(X(s, x_0), l).$$

The CEDE would follow by identifying the state transition vectors l such that $l_p = 1, -1$ for each p and obtaining the corresponding $f(\cdot, l)$ functions from Sec C.3. We undertake that exercise for a few example equations of the CEDE for the "vaccine only" scenario, namely (16) - (31). We choose the "vaccine only" scenario for this illustration because the CEDE for the "both drug and vaccine scenario" had been outlined based on the CEDE for the "vaccine only" scenario, while the CEDE for the "no countermeasure" scenario, namely (1) - (14), is simpler and the CEDE for the "drug only" scenario was outlined based on that for the "no countermeasure" scenario.

We show how (16) of the CEDE for the "vaccine only" scenario follows. Let component p of $X(s, x_0)$ correspond to the fraction of individuals who are susceptibles, immunocompetent, cooperative, and in cluster g. $l_p = 1$ for the transitions representing 1) the movement of immunocompetent, cooperative susceptibles to cluster g; 2) conversion of immunocompetent, non-cooperative susceptibles in cluster g to cooperative ones. $l_p = -1$ for the following transitions involving p: 1) infection; 2) movement away from cluster g; 3) preemption of immunocompetent, cooperative susceptibles in cluster g. Now considering the transitions corresponding to $l_p = 1$, 1) gives us the fourth term in (16) with $f(\cdot)$ obtained from *change location* transition in Sec C.3, 2) gives us the second term in (16) with $f(\cdot)$ obtained from *change opinion* transition and recalling that $X_j(t)$ in (16) represents fraction of individuals in cluster j who are cooperative. Now considering the transitions corresponding to $l_p = -1$, 1) gives us the first term in (16) considering $f(\cdot)$ for *spread infection* transition and recalling that $I_j(t)$ in (16) is the fraction of individuals in cluster j who are infectious, 2) gives us the fifth term in (16) with $f(\cdot)$ obtained from *change location* transition in Sec C.3, 3) gives us the third term in (16) considering $f(\cdot)$ for *preemption* transition in Sec C.3.

Next let component p correspond to the fraction of individuals who are susceptible, immunocompetent, non-cooperative, and in cluster g. We obtain (17) similar to (16) with the following difference. Since non-cooperative individuals cannot be vaccinated, there is no preemption term in (17). Preemption occurs only through vaccination for this CEDE (which is for the "vaccine only" scenario). The terms on the right hand side of (17) can be obtained from the various $f(\cdot)$ functions as in the above paragraph.

Next consider the component p representing the fraction of individuals who are in early incubation, are immunocompetent and in cluster g. We obtain (18) as in the first paragraph with the following differences. Now a *spread infection* transition is represented by a l in which $l_p = 1$ instead of -1. The *disease progression* transition is represented by a l in which $l_p = -1$; the corresponding $f(\cdot)$ can be obtained from Sec C.3 with $\zeta = \omega$ (this specific transition is from early to late incubation). This provides us the fourth term in (18). The rest of the terms in (18) can be obtained as discussed for (16).

(19) can be obtained based on (18) as (17) was obtained based on (16).

We now consider the component p corresponding to cooperatives, immunocompetent, late incubators in cluster g. We obtain (20) as we obtained (18) with the following differences: 1) the pth component of the transition vector for the *spread infection* transition is 0 in this case (as the *spread infection* transition converts susceptibles to early incubators) 2) $l_p = 1$ (it was -1 in (18)) for the transition vector l representing the *disease progression* transition from early incubator to late incubator. Now as argued for (18), the corresponding $f(\cdot)$ can be obtained from Sec C.3 with $\zeta = \omega$. This provides us the first term in (20). Other terms can be obtained as for (16).

Similarly, equations (21) to (31) of the CEDE may be derived. The CEDE for the other scenarios may be derived similarly.

References

25. Istvan Z, Miller K, Joel CS, Peter L. Mathematics of Epidemics on Networks: From Exact to Approximate Models. Springer; 2019.

30. Kurtz TG. Solutions of ordinary differential equations as limits of pure jump Markov processes. Journal of applied Probability. 1970 Apr;7(1):49-58.

43. Szabo F. The linear algebra survival guide: illustrated with Mathematica. Academic Press; 2015 Feb 27.
