# Supplementary material for: Countering the potential re-emergence of a deadly infectious disease—Information warfare, identifying strategic threats, launching countermeasures
Source: PLoS One. 2021 Aug 20;16(8):e0256014. doi: 10.1371/journal.pone.0256014 (PMC8378755; doi:10.1371/journal.pone.0256014)
Supplement: S1 Appendix — (PDF) [file pone.0256014.s002.pdf]

# A Equations of smallpox disease dynamics

## A.1 Developing the Clustered Epidemiological Differential Equations for Disease, Information and Countermeasure Dynamics

We first consider the case of no countermeasure.

**Table 4. Relevant abbreviations (no countermeasure scenario)**

| Compartment | Description |
|---|---|
| $S_c$ | immunodeficient susceptibles |
| $S_h$ | immunocompetent susceptibles |
| $A_c$ | immunodeficient early incubators |
| $A_h$ | immunocompetent early incubators |
| $B_c$ | immunodeficient late incubators |
| $B_h$ | immunocompetent late incubators |
| $P_c$ | immunodeficient prodromals |
| $P_h$ | immunocompetent prodromals |
| $C_c$ | immunodeficient individuals in early rash stage |
| $C_h$ | immunocompetent individuals in early rash stage |
| $E_c$ | immunodeficient individuals in late rash stage |
| $E_h$ | immunocompetent individuals in late rash stage |
| $R$ | Individuals that recovered from the disease |
| $D$ | Dead |

Referring to the notations in Table 4, we index each term by the clusters the individuals in the state inhabit. More specifically, $S_{hi}(t)$ is the fraction of individuals who are susceptible and immunocompetent and are in cluster $i$ at time $t$, $S_{ci}(t), A_{hi}(t), A_{ci}(t), B_{hi}(t), B_{ci}(t), P_{hi}(t), P_{ci}(t), C_{hi}(t), C_{ci}(t)$ may be defined

similarly using Table 4 as a basis. Finally, $R_i(t), D_i(t)$ are respectively the fractions of individuals who are recovered and dead and are in cluster $i$ at time $t$. Let $I_i(t)$ be the fraction of the *infectious* individuals in cluster $i$ at time $t$, that is, the individuals who can pass on the disease to the susceptibles through contact. Then,

$$I_i(t) = P_{ci}(t) + P_{hi}(t) + C_{ci}(t) + C_{hi}(t) + E_{ci}(t) + E_{hi}(t).$$

Interaction between individuals corresponding to $S_{hi}(t), S_{ci}(t)$ and $I_j(t)$ spread the disease to the susceptibles and transform them to early incubators - refer to the yellow arrows in Fig 1. Natural progression of the disease change $A_{hi}(t)$ to $B_{hi}(t)$ to $P_{hi}(t)$ to $C_{hi}(t)$ to $E_{hi}(t)$, and finally $E_{hi}(t)$ to either $R_i(t)$ or $D_i(t)$; also, $A_{ci}(t)$ to $B_{ci}(t)$ to $P_{ci}(t)$ to $C_{ci}(t)$ to $E_{ci}(t)$, and finally $E_{ci}(t)$ to either $R_i(t)$ or $D_i(t)$ - refer to the blue arrows in Fig 1. Mobility changes the cluster indices. The transitions are similar for the immunocompetent and immunodeficient individuals, but the fractions of those who recover in each populace are different.

We model the evolution of the states as per a set of epidemiological differential equations, which we call *clustered epidemiological differential equations* or the CEDE. Let $\dot{S}_{ci}$ be the derivative of $S_{ci}(t)$ with respect to time $t$ (i.e., the rate of change with respect to $t$). $\dot{A}_{hi}(t)$, $\dot{A}_{ci}(t)$, $\dot{B}_{hi}(t)$, $\dot{B}_{ci}(t)$, $\dot{P}_{hi}(t)$, $\dot{P}_{ci}(t)$, $\dot{C}_{hi}(t)$, $\dot{C}_{ci}(t)$, $\dot{R}_i(t)$, $\dot{D}_i(t)$ may be defined similarly. The parameters of the CEDE have been summarized and defined in Tables 5 and 6 next.

**Table 5. Parameters for the CEDE in the no countermeasure scenario**

| Parameter | Symbols | Some associated terms in (1) - (14) |
|---|---|---|
| Mobility rate | $\kappa_{i,j}$ | $S_{hj}(t)\kappa_{ij}, B_{ci}(t)\kappa_{ji}, E_{hj}(t)\kappa_{ij}, P_{ci}(t)\kappa_{ji}, etc.$ |
| Disease spread rate | $\phi_{i,j}$ | $S_{hi}(t)\phi_{ji}I_j(t), S_{ci}(t)\phi_{ji}I_j(t)$ |
| Opinion spread rate | $\alpha_{i,j}$ | This scenario is not affected by opinion |
| Disease progression rate | $\omega, \beta, \gamma, \rho, \delta$ | $A_{hi}(t)\omega, P_{ci}(t)\beta, C_{hi}(t)\rho, R_{ci}(t)\delta$, etc. |

**Table 6. The disease progression rate parameters**

| Parameters | Description of parameters |
|---|---|
| $\frac{1}{\omega}$ | Expected time an individual is in early incubation |
| $\frac{1}{\beta}$ | Expected time an individual is in late incubation |
| $\frac{1}{\gamma}$ | Expected time an individual is in prodrome stage |
| $\frac{1}{\rho}$ | Expected time an individual is in early rash stage |
| $\frac{1}{\delta}$ | Expected time an individual is in late rash stage |
| $\sigma$ | Probability that an immunocompetent individual at late rash stage dies |
| $\tilde{\sigma}$ | Probability that an immunodeficient individual at late rash stage dies |

The CEDE follows:

$$
\begin{aligned}
\dot{S}_{hi}(t) &= -\sum_{j=1}^{n} S_{hi}(t)\phi_{ji}I_j(t) + \sum_{\substack{j=1\\ j\neq i}}^{n} S_{hj}(t)\kappa_{ij} - \sum_{\substack{i=1\\ i\neq j}}^{n} S_{hi}(t)\kappa_{ji} && (1)\\
\dot{S}_{ci}(t) &= -\sum_{j=1}^{n} S_{ci}(t)\phi_{ji}I_j(t) + \sum_{\substack{j=1\\ j\neq i}}^{n} S_{cj}(t)\kappa_{ij} - \sum_{\substack{i=1\\ i\neq j}}^{n} S_{ci}(t)\kappa_{ji} && (2)\\
\dot{A}_{hi}(t) &= \sum_{j=1}^{n} S_{hi}(t)\phi_{ji}I_j(t) - A_{hi}(t)\omega + \sum_{\substack{j=1\\ j\neq i}}^{n} A_{hj}(t)\kappa_{ij} - \sum_{\substack{i=1\\ i\neq j}}^{n} A_{hi}(t)\kappa_{ji} && (3)\\
\dot{A}_{ci}(t) &= \sum_{j=1}^{n} S_{ci}(t)\phi_{ji}I_j(t) - A_{ci}(t)\omega + \sum_{\substack{j=1\\ j\neq i}}^{n} A_{cj}(t)\kappa_{ij} - \sum_{\substack{i=1\\ i\neq j}}^{n} A_{ci}(t)\kappa_{ji} && (4)\\
\dot{B}_{hi}(t) &= A_{hi}(t)\omega - B_{hi}(t)\beta + \sum_{\substack{j=1\\ j\neq i}}^{n} B_{hj}(t)\kappa_{ij} - \sum_{\substack{i=1\\ i\neq j}}^{n} B_{hi}(t)\kappa_{ji} && (5)\\
\dot{B}_{ci}(t) &= A_{ci}(t)\omega - B_{ci}(t)\beta + \sum_{\substack{j=1\\ j\neq i}}^{n} B_{cj}(t)\kappa_{ij} - \sum_{\substack{i=1\\ i\neq j}}^{n} B_{ci}(t)\kappa_{ji} && (6)\\
\dot{P}_{hi}(t) &= B_{hi}(t)\beta - P_{hi}(t)\gamma + \sum_{\substack{j=1\\ j\neq i}}^{n} P_{hj}(t)\kappa_{ij} - \sum_{\substack{i=1\\ i\neq j}}^{n} P_{hi}(t)\kappa_{ji} && (7)\\
\dot{P}_{ci}(t) &= B_{ci}(t)\beta - P_{ci}(t)\gamma + \sum_{\substack{j=1\\ j\neq i}}^{n} P_{cj}(t)\kappa_{ij} - \sum_{\substack{i=1\\ i\neq j}}^{n} P_{ci}(t)\kappa_{ji} && (8)\\
\dot{C}_{hi}(t) &= P_{hi}(t)\gamma - C_{hi}(t)\rho + \sum_{\substack{j=1\\ j\neq i}}^{n} C_{hj}(t)\kappa_{ij} - \sum_{\substack{i=1\\ i\neq j}}^{n} C_{hi}(t)\kappa_{ji} && (9)\\
\dot{C}_{ci}(t) &= P_{ci}(t)\gamma - C_{ci}(t)\rho + \sum_{\substack{j=1\\ j\neq i}}^{n} C_{cj}(t)\kappa_{ij} - \sum_{\substack{i=1\\ i\neq j}}^{n} C_{ci}(t)\kappa_{ji} && (10)\\
\dot{E}_{hi}(t) &= C_{hi}(t)\rho - E_{hi}(t)\delta + \sum_{\substack{j=1\\ j\neq i}}^{n} E_{hj}(t)\kappa_{ij} - \sum_{\substack{i=1\\ i\neq j}}^{n} E_{hi}(t)\kappa_{ji} && (11)\\
\dot{E}_{ci}(t) &= C_{ci}(t)\rho - E_{ci}(t)\delta + \sum_{\substack{j=1\\ j\neq i}}^{n} E_{cj}(t)\kappa_{ij} - \sum_{\substack{i=1\\ i\neq j}}^{n} E_{ci}(t)\kappa_{ji} && (12)\\
\dot{R}_{i}(t) &= ((1-\sigma)E_{hi}(t) + (1-\tilde{\sigma}E_{ci}(t))\,\delta + \sum_{\substack{j=1\\ j\neq i}}^{n} R_{hj}(t)\kappa_{ij} - \sum_{\substack{i=1\\ i\neq j}}^{n} R_{hi}(t)\kappa_{ji} && (13)\\
\dot{D}_{i}(t) &= \tilde{\sigma}E_{ci}(t)\delta + \sigma E_{hi}(t)\delta && (14)
\end{aligned}
$$

First note that (1) is similar to (2) as transitions are similar for the immunocompetent and immunodeficient individuals. The same observation may be made for ((3), (4)), ((5), (6)), ((7), (8)), ((9), (10)), ((11), (12)). So, we only explain (1), (3), (5), (7), (9), (11), as also (13), (14).

The first terms in (1), (3), (the terms in green color) are quadratic terms which represent the spread of the disease to the susceptibles due to interaction with the infectious individuals and the subsequent transformation of the susceptibles to early incubators - refer to the yellow arrows in Fig 1. We provide an intuition for these quadratic terms next. The rate of conversion of susceptibles in a cluster to early incubators is proportional to the number of physical contacts per unit time between the susceptibles in the cluster with infectious individuals, since each such contact spreads the disease to the susceptible with a certain probability. The proportionality constant here is the probability that such a contact spreads the disease or equivalently the fraction of such contacts that spread the disease. Note that in general the infectious and susceptible can be in the same or adjoining clusters. The number of such physical contacts per unit time again is proportional to the number of pairs of susceptibles in the cluster in question and infectious in the same and adjoining clusters. Here the proportionality constant is the reciprocal of the expected time between successive physical contacts between individuals in a given such pair. The expected time will presumably be lower, and therefore the proportionality constant higher, if both individuals are in the same cluster than if they are in different clusters since connections between geographically close individuals are more frequent [1, 2]. Number of pairs of individuals one of which is a susceptible in a cluster $i$ and another an infectious individual in cluster $j$, where $i$, $j$ may be same or different, is the product of the number of susceptibles in $i$ and infectious in $j$. These products lead to the quadratic terms. The overall proportionality constants are the products of the two proportionality constants mentioned above, and give us $\phi_{i,j}$ , the disease spread rates, which have been defined in Section 2.2 and have also been summarized in Table 5. Since the spread of the disease reduces the number of susceptibles and increases the number of early incubators, the first term in (1) has a positive sign and that in (3) has a negative sign.

The last two terms in (1), (3), (5), (7), (9), (11), (13) (the terms in blue) are linear terms representing mobility of individuals across clusters. These linear terms arise because the rate of movement of individuals of a certain type (e.g., susceptible) from a cluster $i$ to another is proportional to the number of individuals of that type in cluster $i$. The proportionality constants constitute the mobility rates which have been defined in Section 2.2 and have also been summarized in Table 5. The positive and negative terms respectively represent inflow into and outflow from the cluster. The dead individuals do not move, so we do not see such

terms in (14).

All other terms in (3), (5), (7), (9), (11), (13), and (14) (the terms in red) represent the natural progression of the disease - refer to the yellow arrows in Fig 1. These linear terms arise because the rate of transition of individuals in one stage of the disease to the next in a cluster $i$ is proportional to the number of individuals in the stage in question in cluster $i$. The positive (negative, respectively) terms correspond to arrows directed towards (away, respectively) from the corresponding state. For example the second term in (3) and the first term in (5) represent the natural progression from early incubation to late incubation. The first term in (13) represents the recovery of immunocompetent and immunodeficient individuals from the late rash stage.

Finally, the solution of the CEDE provides the spatio-temporal distribution for the spread of the disease, namely the fraction of individuals who (1) are dead and are in cluster $i$ at time $t$ ($D_i(t)$), (2) have recovered and are in cluster $i$ at time $t$ ($R_i(t)$), (3) are susceptible and are in cluster $i$ at time $t$ ($(S_{ci} + S_{hi})(t)$), (4) are infected and are in cluster $i$ at time $t$ ($(P_{ci} + P_{hi} + C_{ci} + C_{hi} + E_{ci} + E_{hi})(t)$), (5) are contagious and are in cluster $i$ at time $t$ ($(S_{ci} + S_{hi} + A_{ci} + A_{hi} + B_{ci} + B_{hi} + P_{ci} + P_{hi} + C_{ci} + C_{hi} + E_{ci} + E_{hi})(t)$).

## A.2 Drug only

Comparing the state transitions for the “no countermeasure” and the “drug only” scenarios, as depicted in Figs 1 and 2, we notice that the latter varies from the former in that it has one additional state called *Preempted* or $Q$. Thus, the CEDE for the “drug only” scenario can be obtained from (1) to (14) by adding an equation that models the transition to this state and modifying (1) to (14) to reflect this transition. Let $Q(t)$ be the fraction of individuals who have been preempted after receiving the drug. Then, (15) represents the evolution of $Q(t)$.

$$\begin{aligned}\dot{Q}(t) \quad = \quad & \sum_{i=1}^{n} (P_{ci}(t)\tau_i + P_{hi}(t)\tau_i + C_{ci}(t)\mu_i + C_{hi}(t)\mu_i + B_{ci}(t)\tau_i \\ & + B_{hi}(t)\tau_i + A_{ci}(t)\tau_i + A_{hi}(t)\tau_i + S_{ci}(t)\tau_i + S_{hi}(t)\tau_i)\end{aligned} \tag{15}$$

Here $\tau_i$ is the reciprocal of the expected time required for an individual to receive the drug for the first time in cluster $i$ times the preemption probability given that he is yet to reach the rash stage. Similarly, $\mu_i$ is the reciprocal of the expected time required for patients in cluster $i$ to receive the drug times the preemption

probability in rash stage. Preemption probability for drug treatment is the probability that while receiving the drug the individual remains immune to the disease (i.e., does not develop the disease even upon receiving the virus from an infectious contact) and is cured of the disease if he had the disease when he started the drug treatment.

The terms $\tau_i P_{ci}(t)$, $\tau_i P_{hi}(t)$, $\mu_i C_{ci}(t)$, $\mu_i C_{hi}(t)$, $\tau_i A_{ci}(t)$, $\tau_i A_{hi}(t)$, $\tau_i B_{ci}(t)$, $\tau_i B_{hi}(t)$, $\tau_i S_{ci}(t)$, $\tau_i S_{hi}(t)$, represent the preemption of the individuals in cluster $i$ in the respective states. Due to these preemptions, (1) through (13) will respectively be decremented by $S_{hi}\tau_i$, $S_{ci}\tau_i$, $A_{hi}\tau_i$, $A_{ci}\tau_i$, $B_{hi}\tau_i$, $B_{ci}\tau_i$, $P_{hi}\tau_i$, $P_{ci}\tau_i$, $C_{hi}\mu_i$, and $C_{ci}\mu_i$.

The CEDE above can cater to a range of policies for administering of the drug through appropriate choice of the $\tau_i$, and $\mu_i$. A policy choice may for example be to administer the drug at a higher rate in certain clusters, either because of their centrality and thereby the prospect of infectious individuals therein infecting others at a higher rate, or because the number of infected individuals is high there. Such policies may be represented by selecting a higher value for the corresponding $\tau_i$s. A policy choice may be to administer the drug to only those showing certain symptoms, e.g., fever. The $\tau_i$s may then be selected based on the stage of the disease - then they would be indexed by the stage as well. Finally, when the drug availability is finite, $\tau_i$ and $\mu_i$ may be made dependent on time, and as the drug supply is exhausted, the $\tau_i$ and $\mu_i$ may be chosen to be 0.

Finally, the solution of the CEDE provides the spatio-temporal distribution for the spread of the disease and the impact of the countermeasure (drug). These are captured in the fraction of individuals who (1) are dead and are in cluster $i$ at time $t$ ($D_i(t)$), (2) have recovered and are in cluster $i$ at time $t$ ($R_i(t)$), (3) are preempted (through drug) and are in cluster $i$ at time $t$ ($Q_i(t)$), (4) are susceptible and are in cluster $i$ at time $t$ ($(S_{ci} + S_{hi})(t)$), (5) are infected and are in cluster $i$ at time $t$ ($(P_{ci} + P_{hi} + C_{ci} + C_{hi} + E_{ci} + E_{hi})(t)$), (6) are contagious and are in cluster $i$ at time $t$
$((S_{ci} + S_{hi} + A_{ci} + A_{hi} + B_{ci} + B_{hi} + P_{ci} + P_{hi} + C_{ci} + C_{hi} + E_{ci} + E_{hi})(t))$.

## A.3 Vaccine only

The principal difference between the "Vaccine only" scenario and the earlier scenarios lies in the incorporation of the opinion dynamics. The individuals must now be classified based on their cooperativity, leading to the new states enunciated in Table 7.

**Table 7. Relevant abbreviations (vaccine only scenario)**

| Compartment | Description |
|---|---|
| $S_{ac}$ | immunodeficient cooperative susceptibles |
| $S_{bc}$ | immunodeficient non-cooperative susceptibles |
| $S_{ah}$ | immunocompetent cooperative susceptibles |
| $S_{bh}$ | immunocompetent non-cooperative susceptibles |
| $A_{ac}$ | immunodeficient cooperative early incubators |
| $A_{bc}$ | immunodeficient non-cooperative early incubators |
| $A_{ah}$ | immunocompetent cooperative early incubators |
| $A_{bh}$ | immunocompetent non-cooperative early incubators |
| $B_{ac}$ | immunodeficient cooperative late incubators |
| $B_{bc}$ | immunodeficient non-cooperative late incubators |
| $B_{ah}$ | immunocompetent cooperative late incubators |
| $B_{bh}$ | immunocompetent non-cooperative late incubators |
| $P_{ac}$ | immunodeficient cooperative prodromals |
| $P_{bc}$ | immunodeficient non-cooperative prodromals |
| $P_{ah}$ | immunocompetent cooperative prodromals |
| $P_{bh}$ | immunocompetent non-cooperative prodromals |
| $C_{ac}$ | immunodeficient cooperative individuals in early rash stage |
| $C_{bc}$ | immunodeficient non-cooperative individuals in early rash stage |
| $C_{ah}$ | immunocompetent cooperative individuals in early rash stage |
| $C_{bh}$ | immunocompetent non-cooperative individuals in early rash stage |
| $E_{ac}$ | immunodeficient cooperative individuals in late rash stage |
| $E_{bc}$ | immunodeficient non-cooperative individuals in late rash stage |
| $E_{ah}$ | immunocompetent cooperative individuals in late rash stage |
| $E_{bh}$ | immunocompetent non-cooperative individuals in late rash stage |
| $R_a$ | Cooperative individuals that recovered from the disease |
| $R_b$ | Non-cooperative individuals that recovered from the disease |
| $Q$ | Vaccinated (immunized) |
| $D$ | Dead |

To consider the impact of space, we further classify individuals in the states given in Table 7 based on the cluster they inhabit. Thus, $S_{ahi}(t)$ ($S_{bci}(t)$, respectively) is the fraction of immunocompetent cooperative (immunodeficient non-cooperative, respectively) susceptibles who are in cluster $i$ at time $t$. As before, $I_i(t)$ is the fraction of the *infectious* individuals in cluster $i$ at time $t$. Let $X_i(t)$ be the fraction of individuals who are cooperative and are in cluster $i$ at time $t$. Thus,

$$\begin{aligned}
I_i(t) &= P_{ahi}(t) + P_{aci}(t) + P_{bhi}(t) + P_{bci}(t) + C_{ahi}(t) + C_{aci}(t) + C_{bhi}(t) + C_{bci}(t) \\
&\quad + E_{ahi}(t) + E_{aci}(t) + E_{bhi}(t) + E_{bci}(t) \\
X_i(t) &= V_i(t) + S_{ahi}(t) + S_{aci}(t) + A_{ahi}(t) + A_{aci}(t) + B_{ahi}(t) + B_{aci}(t) + P_{ahi}(t) \\
&\quad + P_{aci}(t) + C_{ahi}(t) + C_{aci}(t) + E_{ahi}(t) + E_{aci}(t) + R_{ahi}(t) + R_{aci}(t)
\end{aligned}$$

The spread of opinions is modeled in the CEDE through quadratic terms (the terms in orange) representing opinion exchange interactions between the non-cooperative and the cooperative individuals, just as the spread of disease is modeled through quadratic terms (the terms in green) representing physical contact based interactions between the susceptible and infectious individuals. For simplicity, in this subsection, we present the CEDE only for the scenario that during opinion exchanges the cooperatives persuade the noncooperatives to become cooperatives.

The immunocompetent cooperative susceptibles (respectively early incubators) who are in cluster $i$ are preempted (through vaccination) at the rate $\theta_i$ ($\pi_i$ respectively). The preemption rate is the product of the reciprocal of the expected delay in developing immunity and the preemption probability. The preemption probability is the probability that the vaccine raises the immunity level of the recipient such that he does not develop the disease, from the virus he may receive from an infectious contact in future or from the virus he has already received if he is an early incubator. The expected delay is the sum of the expected delays in 1) receiving the vaccine (e.g., delay incurred in getting the health worker's appointment) and 2) developing immunity after receiving the vaccine. After these individuals are vaccinated and develop immunity, they transition to preempted state $Q$ (the terms in black in the CEDE below); this process is denoted by the yellow arrows in Fig 3. The fraction of individuals who are vaccinated and are in cluster $i$ at time $t$ is $Q_i(t)$.

The resulting CEDE, for the evolution of the states of the immunocompetent individuals, is shown in (16) - (31) with the coefficients defined in Tables 5 and 6 and additional parameters defined in Table 8.

**Table 8. Symbols for rate of preemption (due to vaccination) in the "vaccine only" scenario**

| Symbols | Qualifications |
|---|---|
| $\theta$ | Rates for immunocompetent cooperative susceptibles |
| $\tilde{\theta}$ | Rates for immunodeficient cooperative susceptibles |
| $\pi$ | Rates for immunocompetent cooperative early incubators |
| $\tilde{\pi}$ | Rates for immunodeficient cooperative early incubators |

$$\dot{S}_{ahi}(t) = -\sum_{j=1}^{n} S_{ahi}(t)\phi_{ji}(t)I_j(t) + \sum_{j=1}^{n} S_{bhi}(t)\alpha_{ji}X_j(t) - S_{ahi}(t)\theta_i + \sum_{\substack{j=1\\ j\neq i}}^{n} S_{ahj}(t)\kappa_{ij} - \sum_{\substack{i=1\\ i\neq j}}^{n} S_{ahi}(t)\kappa_{ji} \quad (16)$$

$$\dot{S}_{bhi}(t) = -\sum_{j=1}^{n} S_{bhi}(t)\phi_{ji}I_j(t) - \sum_{j=1}^{n} S_{bhi}(t)\alpha_{ji}X_j(t) + \sum_{\substack{j=1\\ j\neq i}}^{n} S_{bhj}(t)\kappa_{ij} - \sum_{\substack{i=1\\ i\neq j}}^{n} S_{bhi}(t)\kappa_{ji} \quad (17)$$

$$\dot{A}_{ahi}(t) = \sum_{j=1}^{n} S_{ahi}(t)\phi_{ji}I_j(t) + \sum_{j=1}^{n} A_{bhi}(t)\alpha_{ji}X_j(t) - A_{ahi}(t)\pi_i - A_{ahi}(t)\omega + \sum_{\substack{j=1\\ j\neq i}}^{n} A_{ahj}(t)\kappa_{ij} - \sum_{\substack{i=1\\ i\neq j}}^{n} A_{ahi}(t)\kappa_{ji} \quad (18)$$

$$\dot{A}bh_i(t) = \sum_{j=1}^{n} S_{bhi}(t)\phi_{ji}I_j(t) - \sum_{j=1}^{n} A_{bhi}(t)\alpha_{ji}X_j(t) - A_{bhi}(t)\omega + \sum_{\substack{j=1\\ j\neq i}}^{n} A_{bhj}(t)\kappa_{ij} - \sum_{\substack{i=1\\ i\neq j}}^{n} A_{bhi}(t)\kappa_{ji} \quad (19)$$

$$\dot{B}_{ahi}(t) = A_{ahi}(t)\omega - B_{ahi}(t)\beta_i + \sum_{j=1}^{n} B_{bhi}(t)\alpha_{ji}X_j(t) + \sum_{\substack{j=1\\ j\neq i}}^{n} B_{ahj}(t)\kappa_{ij} - \sum_{\substack{i=1\\ i\neq j}}^{n} B_{ahi}(t)\kappa_{ji} \quad (20)$$

$$\dot{B}_{bhi}(t) = A_{bhi}(t)\omega - B_{bhi}(t)\beta - \sum_{j=1}^{n} B_{bhi}(t)\alpha_{ji}X_j(t) + \sum_{\substack{j=1\\ j\neq i}}^{n} B_{bhj}(t)\kappa_{ij} - \sum_{\substack{i=1\\ i\neq j}}^{n} B_{bhi}(t)\kappa_{ji} \quad (21)$$

$$\dot{P}_{ahi}(t) = B_{ahi}(t)\beta - P_{ahi}(t)\gamma + \sum_{j=1}^{n} P_{bhi}(t)\alpha_{ji}X_j(t) + \sum_{\substack{j=1\\ j\neq i}}^{n} P_{ahj}(t)\kappa_{ij} - \sum_{\substack{i=1\\ i\neq j}}^{n} P_{ahi}(t)\kappa_{ji} \quad (22)$$

$$\dot{P}_{bhi}(t) = B_{bhi}(t)\beta - P_{bhi}(t)\gamma - \sum_{j=1}^{n} P_{bhi}(t)\alpha_{ji}X_j + \sum_{\substack{j=1\\ j\neq i}}^{n} P_{bhj}(t)\kappa_{ij} - \sum_{\substack{i=1\\ i\neq j}}^{n} P_{bhi}(t)\kappa_{ji} \quad (23)$$

$$\dot{C}_{ahi}(t) = P_{ahi}(t)\gamma - C_{bhi}(t)\rho + \sum_{j=1}^{n} C_{bhi}(t)\alpha_{ji}X_j + \sum_{\substack{j=1\\ j\neq i}}^{n} C_{bhj}(t)\kappa_{ij} - \sum_{\substack{i=1\\ i\neq j}}^{n} C_{bhi}(t)\kappa_{ji} \quad (24)$$

$$\dot{C}_{bhi}(t) = P_{bhi}(t)\gamma - C_{bhi}(t)\rho - \sum_{j=1}^{n} C_{bhi}(t)\alpha_{ji}X_j + \sum_{\substack{j=1\\ j\neq i}}^{n} C_{bhj}(t)\kappa_{ij} - \sum_{\substack{i=1\\ i\neq j}}^{n} C_{bhi}(t)\kappa_{ji} \quad (25)$$

$$\dot{E}_{ahi}(t) = C_{ahi}(t)\rho - E_{ahi}(t)\delta + \sum_{j=1}^{n} E_{bhi}(t)\alpha_{ji}X_j(t) + \sum_{\substack{j=1\\ j\neq i}}^{n} E_{ahj}(t)\kappa_{ij} - \sum_{\substack{i=1\\ i\neq j}}^{n} E_{ahi}(t)\kappa_{ji} \quad (26)$$

$$\dot{E}_{bhi}(t) = C_{bhi}(t)\rho - E_{bhi}(t)\delta - \sum_{j=1}^{n} E_{bhi}(t)\alpha_{ji}X_j(t) + \sum_{\substack{j=1\\ j\neq i}}^{n} E_{bhj}(t)\kappa_{ij} - \sum_{\substack{i=1\\ i\neq j}}^{n} E_{bhi}(t)\kappa_{ji} \quad (27)$$

$$\dot{R}_{ahi}(t) = (1-\sigma)E_{ahi}(t)\delta + \sum_{j=1}^{n} R_{bhi}(t)\alpha_{ji}X_j(t) + \sum_{\substack{j=1\\ j\neq i}}^{n} R_{ahj}(t)\kappa_{ij} - \sum_{\substack{i=1\\ i\neq j}}^{n} R_{ahi}(t)\kappa_{ji} \quad (28)$$

$$\dot{R}_{bhi}(t) = (1-\sigma)E_{bhi}(t)\delta + \sum_{j=1}^{n} R_{bhi}(t)\alpha_{ji}X_j + \sum_{\substack{j=1\\ j\neq i}}^{n} R_{bhj}(t)\kappa_{ij} - \sum_{\substack{i=1\\ i\neq j}}^{n} R_{bhi}(t)\kappa_{ji} \quad (29)$$

$$\dot{Q}_i(t) = S_{ahi}(t)\theta_i + S_{aci}(t)\tilde{\theta}_i + A_{ahi}(t)\pi_i + A_{aci}(t)\tilde{\pi}_i + \sum_{\substack{j=1\\ j\neq i}}^{n} Q_{bhj}(t)\kappa_{ij} - \sum_{\substack{i=1\\ i\neq j}}^{n} Q_{bhi}(t)\kappa_{ji} \quad (30)$$

$$\dot{D}_i(t) = \sigma E_{ahi}(t)\delta + \tilde{\sigma}E_{aci}(t)\delta + \sigma E_{bhi}(t)\delta + \tilde{\sigma}E_{bci}(t)\delta \quad (31)$$

The differential equations for the evolution of the states of the immunodeficient individuals is identical to those of the immunocompetent individuals, as seen in equations (1) to (12) in Appendix A.1. Specifically, the equation for $\dot{S}_{aci}(t)$ may be obtained from equation (16) for $\dot{S}_{ahi}(t)$, as the equation (2) for $\dot{S}_{ci}(t)$ followed from equation (1) for $\dot{S}_{hi}(t)$. The only differences in the equations for the immunodeficient individuals are that 1) they have lower preemption rates than the immunocompetent ones as they can avail of different vaccines that act slower and 2) those in late rash stage die while the corresponding immunocompetent ones

can recover. Specifically, the immunodeficient cooperative susceptibles (respectively early incubators) that are in cluster $i$ develop immunity at rate $\tilde{\theta}_i$ ($\tilde{\pi}_i$ respectively). The preemption rates are defined as for the immunocompetent ones, but have lower value as the immunodeficient ones can only receive vaccines that provide immunity after multiple shots administered after specified gaps. Thus, in general, $\tilde{\theta}_i < \theta_i$ and $\tilde{\pi}_i < \pi_i$. Note that the recovery and death rates for the immunodeficient and the immunocompetent ones are as in Table 6.

We now explain the terms in (16) - (31). The second terms in (16) through (29) (the terms in orange) are quadratic terms which represent the transformation of non-cooperative individuals to cooperative ones through exchange of opinion with cooperative individuals - refer to the black arrows in Fig 3. Note that only a fraction of the opinion exchanges between cooperatives and noncooperatives transform the latter. Thus the rate of this transformation for noncooperatives of a certain type (the type is specified by the stage of disease, cluster, immunocompetence/immunodeficiency) is proportional to the number of opinion exchanges per unit time between the noncooperatives of that type with cooperatives (in the same or in adjoining clusters); the proportionality constant is the value of the above fraction. The number of such exchanges per unit time again is proportional to the number of pairs of noncooperatives of the type in question and cooperatives in the same and adjoining clusters; the proportionality constant is the reciprocal of the average time between successive interactions of a given pair. Number of pairs of individuals one of which is a noncooperative of a certain type and another a cooperative in the same or adjoining cluster, is the product of the number of noncooperatives of the type and cooperatives in the same or adjoining clusters. These products lead to the quadratic terms. The overall proportionality constant is the product of the two proportionality constants above, and provide us the opinion spread rates, $\alpha_{i,j}$, which have been defined in Section 2.2 and have also been summarized in Table 5. Since the change of opinion increases (respectively decreases) the cooperatives (respectively noncooperatives), the second terms in the equations for the cooperatives (e.g., (16)) are positive and the second terms for the non-cooperatives (e.g., (17)) are negative.

The terms $\theta_i S_{ahi}(t)$ and $\pi_i A_{ahi}(t)$ (the terms in black) in (30) represent the vaccination of immunocompetent cooperative susceptibles and early incubators. Intuitively, these linear terms arise because the number of susceptibles (early incubators, respectively) vaccinated in a cluster per unit time is proportional to the number of susceptibles (early incubators, respectively) in the cluster. The proportionality constants are the rates of preemption due to vaccination, namely $\theta_i$ ($\pi_i$, respectively) for cluster $i$ (Table 8). Vaccination reduces the number of susceptibles and early incubators by transforming them to the vaccinated

state. Thus, these terms are subtracted from (16) and (18) respectively and added to (30). The second and fourth terms of equation (30) correspond to the immunization of immunodeficient cooperative susceptibles and early incubators.

The rest of the terms represent the phenomena modeled in equations (1) to (14) in Appendix A.1. Specifically, the first terms in (16), and (18) (the terms in green) are quadratic terms which represent the spread of the disease to the susceptibles due to interaction with the infectious individuals and the subsequent transformation of the susceptibles to early incubators - refer to the yellow arrows in Fig 3. The disease dynamics is similar for the cooperatives and non-cooperatives. The last two terms in (16), (18), (20), (22), (24), (26), (28) (the terms in blue) are linear terms representing mobility of individuals across clusters. The dead individuals do not move, so we do not see such terms in (31). All other terms in (16), (18), (20), (22), (24), (26) (the terms in red) represent the natural progression of the disease - refer to the blue arrows in Fig 3.

Different types of vaccines and immunization policies may be captured through appropriate choices of the values of $\theta_i, \tilde{\theta}_i, \pi_i, \tilde{\pi}_i$. A policy choice may for example be to administer the vaccine at a higher rate in certain clusters. Such policies may be represented by selecting a higher value for the corresponding parameters. Some vaccines may act faster than others leading again to higher values for the above parameters. Finally, when the availability of the vaccine is finite, the values of these parameters may be made dependent on time, and as the supply of the vaccine is exhausted, the parameters may be chosen to be 0.

Finally, the solution of the CEDE provides the spatio-temporal distribution for the spread of the disease and opinion and the impact of the countermeasure (vaccine). These are captured in the fraction of individuals who (1) are dead and are in cluster $i$ at time $t$ ($D_i(t)$), (2) have recovered and are in cluster $i$ at time $t$ ($(R_{ahi} + R_{bhi})(t)$), (3) are preempted (that is, vaccinated) and are in cluster $i$ at time $t$ ($Q_i(t)$), (4) are susceptible and are in cluster $i$ at time $t$ ($(S_{aci} + S_{ahi} + S_{bci} + S_{bhi})(t)$), (5) are infected and are in cluster $i$ at time $t$ ($(P_{aci} + P_{ahi} + P_{bci} + P_{bhi} + C_{aci} + C_{ahi} + C_{bci} + C_{bhi} + E_{aci} + E_{ahi} + E_{bci} + E_{bhi})(t)$), (6) are contagious and are in cluster $i$ at time $t$ ($(S_{aci} + S_{ahi} + S_{bci} + S_{bhi} + A_{aci} + A_{ahi} + A_{bci} + A_{bhi} + B_{aci} + B_{ahi} + B_{bci} + B_{bhi} + P_{aci} + P_{ahi} + P_{bci} + P_{bhi} + C_{aci} + C_{ahi} + C_{bci} + C_{bhi} + E_{aci} + E_{ahi} + E_{bci} + E_{bhi})(t)$).

## A.4 Both drug and vaccine

In this case, individuals may be preempted through drug or vaccine. Thus, a preempted individual may be either cooperative or non-cooperative. Thus, we classify the preempted individuals into cooperatives, $Q_a$ and non-cooperatives, $Q_b$ (Fig 4). The cooperatives among the susceptibles and early incubators can transition to

the state $Q_a$ after developing immunity through drug or vaccine. Transition from the rest of the states (other than recovered or dead) to $Q_a$ or $Q_b$ occurs through reception of the drug. $Q_a, Q_b$ needs to be indexed by the clusters the individuals inhabit.

**Table 9. Parameters description (both drug and vaccine)**

| Parameters | Description of parameters |
|---|---|
| $\Theta$ | Rate of preemption for immunocompetent cooperative susceptibles through the reception of the drug or the vaccine |
| $\tilde{\Theta}$ | Rate of preemption for immunodeficient cooperative susceptibles through the reception of the drug or the vaccine |
| $\Pi$ | Rate of preemption for immunocompetent cooperative early incubators through the reception of the drug or the vaccine. |
| $\tilde{\Pi}$ | Rate of preemption for immunodeficient early incubators through the reception of the drug or the vaccine. |
| $\bar{\Theta}$ | Rate of preemption for immunocompetent non-cooperative susceptibles through the reception of the drug or the vaccine |
| $\hat{\Theta}$ | Rate of preemption for immunodeficient non-cooperative susceptibles through the reception of the drug or the vaccine |
| $\bar{\Pi}$ | Rate of preemption for immunocompetent non-cooperative early incubators through the reception of the drug or the vaccine. |
| $\hat{\Pi}$ | Rate of preemption for immunodeficient non-cooperative early incubators through the reception of the drug or the vaccine. |
| $\tau$ | Rate of preemption for prodromals and non-cooperatives among susceptibles through reception of drugs |
| $\mu$ | Rate of preemption for patients with rash through reception of drugs. |

We use the parameters as defined in Appendix A.3 with some of the modifications described in Table 9. Thus, the CEDE for "both drug and vaccine" scenario can be obtained from (16) to (29) by adding two equations, (32) and (33) below, that model the transitions to $Q_a, Q_b$ and modifying (16) to (29) to reflect

this transition and the distinction between $Q_a, Q_b$.

$$\dot{Q}_{ai}(t) = \sum_{i=1}^{n} \Big( S_{ahi}(t)\Theta_i + S_{aci}(t)\tilde{\Theta}_i + A_{ahi}(t)\Pi_i + A_{aci}(t)\tilde{\Pi}_i + P_{aci}(t)\tau_i + P_{ahi}(t)\tau_i + C_{aci}(t)\mu_i + C_{ahi}(t)\mu_i + B_{aci}(t)\tau_i + B_{ahi}(t)\tau_i + A_{aci}(t)\tau_i + A_{ahi}(t)\tau_i \Big) \tag{32}$$

$$\dot{Q}_{bi}(t) = \sum_{i=1}^{n} \Big( S_{bci}(t)\hat{\Theta}_i + S_{bhi}(t)\bar{\Theta}_i + A_{bci}(t)\hat{\Pi}_i + A_{bhi}(t)\bar{\Pi}_i + P_{bci}(t)\mu_i + P_{bhi}(t)\mu_i + C_{bci}(t)\mu_i + C_{bhi}(t)\mu_i + B_{bci}(t)\tau_i + B_{bhi}(t)\tau_i + A_{bci}(t)\tau_i + A_{bhi}(t)\tau_i \Big) \tag{33}$$

We now describe the modifications in (16) to (29). The expression for $X_i(t)$ in Appendix (A.3) need to have $Q_{ai}(t)$ instead of $Q_i(t)$. The terms $\Theta_i S_{ahi}(t)$, $\tilde{\Theta}_i S_{aci}(t)$, $\pi_i A_{ahi}(t)$, $\tilde{\pi}_i A_{aci}(t)$, $S_{bci}(t)\hat{\Theta}_i$, $S_{bhi}(t)\bar{\Theta}_i$, $A_{bci}(t)\hat{\Pi}_i$, and $A_{bhi}(t)\bar{\Pi}_i$ represent the preemption of individuals by either vaccine or drugs for individuals in cluster $i$ in their respective states. In addition, $\tau_i P_{ahi}(t)$, $\tau_i P_{aci}(t)$, $\tau_i P_{bhi}(t)$, $\tau_i P_{bci}(t)$, $\mu_i C_{ahi}(t)$, $\mu_i C_{aci}(t)$, $\mu_i C_{bhi}(t)$, $\mu_i C_{bci}(t)$, $\mu_i E_{ahi}(t)$, $\mu_i E_{aci}(t)$, $\tau_i B_{ahi}(t)$, $\tau_i B_{aci}(t)$, $\tau_i B_{bhi}(t)$, and $\tau_i B_{bci}(t)$ represent preemption by drug only for individuals in cluster $i$ in the respective states. Due to these preemptions, (16) through (27) will respectively be decremented by $S_{ah_i}\Theta_i$, $S_{bh_i}\hat{\Theta}_i$, $S_{aci}(t)\tilde{\Theta}_i$, $S_{bci}(t)\hat{\Theta}_i$, $A_{ahi}\Pi_i$, $A_{bci}(t)\hat{\Pi}_i$, $A_{aci}(t)\tau_i$, $A_{bhi}(t)\bar{\Pi}_i$, $B_{ah_i}\tau_i$, $B_{bh_i}\tau_i$, $P_{ah_i}\tau_i$, $P_{bh_i}\tau_i$, $C_{ah_i}\mu_i$, and $C_{bh_i}\mu_i$.

Here the suffixes $a, b$ represent the two opinions. Those with suffix $a$ convert those with suffix $b$. To capture the scenario that cooperatives convert the non-cooperatives, we consider those with suffix $a$ as cooperative, and those with suffix $b$ as non-cooperative. To capture the scenario that non-cooperatives convert the cooperatives, we reverse the designations, that is, those with suffix $a$ are non-cooperatives, and those with suffix $b$ are cooperatives. We now describe how the functional roles of the states with suffix "a" ("b", respectively) can be switched to non-cooperatives (cooperatives, respectively). Note that functionally the difference between the cooperatives and non-cooperatives is that the former opt for vaccination while the latter do not. The vaccinations can be effective only if the individuals are susceptible or in early incubation stage. Thus, in these states, the cooperatives can be preempted through either drugs or vaccines, while the non-cooperatives can only be preempted through drugs (that is, if they are eligible for drugs depending on the administering policies). Thus, the preemption rates of the cooperatives are different from the non-cooperatives only in these stages, and that is the only functional difference. In all other stages the individuals are preempted only through drugs and therefore their preemption rates are the same regardless of whether they are cooperatives or non-cooperatives. Thus, only the preemption rates of the cooperative, i.e.,

suffix-"a", susceptibles (respectively, early incubators) need to be interchanged with the corresponding non-cooperative, i.e., suffix-"b", susceptibles (respectively, early incubators). That is, values of $\Theta$, $\bar{\Theta}$ need to be exchanged. Similarly, values of 1) $\tilde{\Theta}$, $\hat{\Theta}$, 2) $\Pi$, $\bar{\Pi}$, 3) $\tilde{\Pi}$, $\hat{\Pi}$ need to be exchanged. We describe how the preemption rates are chosen in Section B of Appendix.

Finally, various policy choices for countermeasure application may be incorporated through appropriate selection of the parameters in Table 9, similar to the connections made in the concluding paragraphs of Appendices A.2 and A.3. In addition, the solution of the CEDE provides the spatio-temporal distribution for the spread of the disease and opinion and the impact of the countermeasure (vaccine and drug). These are captured in fraction of individuals who (1) are dead and are in cluster $i$ at time $t$ ($D_i(t)$), (2) have recovered and are in cluster $i$ at time $t$ ($(R_{ahi} + R_{bhi})(t)$), (3) are preempted (that is, vaccinated or administered drugs) and are in cluster $i$ at time $t$ ($(Q_{ai} + Q_{bi})(t)$), (4) are susceptible and are in cluster $i$ at time $t$ ($(S_{aci} + S_{ahi} + S_{bci} + S_{bhi})(t)$), (5) are infected and are in cluster $i$ at time $t$ ($(P_{aci} + P_{ahi} + P_{bci} + P_{bhi} + C_{aci} + C_{ahi} + C_{bci} + C_{bhi} + E_{aci} + E_{ahi} + E_{bci} + E_{bhi})(t)$), (6) are contagious and are in cluster $i$ at time $t$ ($(S_{aci} + S_{ahi} + S_{bci} + S_{bhi} + A_{aci} + A_{ahi} + A_{bci} + A_{bhi} + B_{aci} + B_{ahi} + B_{bci} + B_{bhi} + P_{aci} + P_{ahi} + P_{bci} + P_{bhi} + C_{aci} + C_{ahi} + C_{bci} + C_{bhi} + E_{aci} + E_{ahi} + E_{bci} + E_{bhi})(t)$).

Now consider the generalization in which both cooperatives and noncooperatives can convert each other during an interaction. Specifically, each interaction between a cooperative and a noncooperative would convert the former with a probability $p_1$, the latter with a probability $p_2$ and convert neither with a probability $1 - p_1 - p_2$. Now, recall that the opinion spread rate $\alpha_{i,j}$ associated with the orange quadratic terms representing the conversion of opinions in (16) - (29) (which correspond to the state transitions represented by the black arrows in Figs 3 and 4) is the product of the rate of interaction between pairs of individuals with different opinions and the probability of conversion in each interaction. This probability needs to be replaced with $p_1 - p_2$. So, if $p_1 > p_2$, interactions convert cooperatives on an aggregate basis, if $p_1 < p_2$ the reverse happens.

# B Parameter estimation

## B.1 Estimation of parameters used in all the scenarios

We first estimate the parameters that occur in all four scenarios ((1) no countermeasure (2) drug only (3) vaccine only (4) both drug and vaccine) we have considered. We first start with the disease progression

parameters. These parameters have been described in Table 6. Typically, the early incubation phase for smallpox lasts for 7 days and the late incubation phase for 5 days [3]. Assuming that each phase has an exponentially distributed random duration, $1/\omega$, $1/\beta$ are the expected durations in these two phases. Thus, $\omega = 1/7$, $\beta = 1/5$. Typically a patient is in prodromal phase for 3 days [3, 4]. Thus, as for the incubation phases, $\gamma = 1/3$. Typically, the early and late rash periods last for 3 and 7 days respectively [3]. Therefore, as before, $\rho = 1/3$, $\delta = 1/7$. On average, 30% of the infected immunocompetent individuals die of the disease [3, 4]. Thus, $\sigma = 0.3$. According to [5], immunodeficient individuals are not expected to recover from smallpox. Thus, $\tilde{\sigma} = 1$.

We now compute the disease spread rate described in Table 5. We assume $\phi_{i,j} = 0$ if $i \neq j$, that is, there is no direct physical contact and therefore no direct spread of disease between people inhabiting different clusters. The *basic reproductive number*, $R_0$, is defined as the average number of secondary infections caused by a single typical infected individual among a completely susceptible population. [3, 6] estimated $R_0$ for smallpox to be 6.9. We estimate $\phi_{i,i}$ to obtain $R_0 = 6.9$. Following the methodology in [7, 8], we get $\phi_{i,i} = 0.00173$.

Mobility rate and opinion spread rate have been described in Section 2.2 and Table 5. These rates heavily depend on the ambience. Therefore, in our numerical computations, we vary these across a range of values, rather than estimating specific values. Note that opinion spread rate does not appear in "no countermeasure" and "drug only" scenarios.

## B.2 Estimation of parameters related to delivery of drugs

We now estimate the parameters related to the preemption of infected individuals by drugs (refer to Appendix A.2), these will also be used to compute the parameters in "both drug and vaccine" scenarios. First, note that the stage of the disease in which an individual can be administered a drug depends on the drug administering policy. We assume that in a state in which an individual is allowed to receive a drug, he needs to wait for an exponentially distributed random time after which he is preempted, that is, he is administered the drug and develops the ability to thwart the disease. Let $\varepsilon$ be the parameter of this exponential process. We also assumed that the wait time for different individuals is independent.

Let there be $v$ individuals in all. The first individual who is preempted waits for the minimum time among $v$. From the theory of exponential distributions, the minimum time is exponentially distributed with parameter $v\varepsilon$, and has expectation $1/v\varepsilon$. The second person to be preempted has the minimum additional

wait time among the remaining $v-1$. Similarly, this additional wait time is exponentially distributed with parameter $(v-1)\varepsilon$ and expectation $1/(v-1)\varepsilon$. Similarly, the additional wait time for the third person (after the second person is preempted) is exponential with parameter $(v-2)\varepsilon$, and has expectation $\frac{1}{(v-2)\varepsilon}$. This continues until the last person whose expected additional wait time is $\frac{1}{\varepsilon}$. Therefore, the total expected time for n individuals to be preempted is,

$$\frac{1}{v\varepsilon}+\frac{1}{(v-1)\varepsilon}+\frac{1}{(v-2)\varepsilon}+...+\frac{1}{\varepsilon}$$

$$=\frac{1}{\varepsilon}\left[\frac{1}{v}+\frac{1}{v-1}+\frac{1}{v-2}+\frac{1}{v-3}+...+\frac{1}{3}+\frac{1}{2}+1\right]$$

$$=\frac{1}{\varepsilon}\sum_{k=1}^{v}\frac{1}{k}\approx\frac{1}{\varepsilon}\left(ln(v)+constant\right)$$

The last expression follows from the Harmonic series (number) approximation. We obtain $\varepsilon$ by equating this expression to the total wait times required to develop immunity as reported in the literature for cities. [3] and [9] state that the CDC's Cities Readiness Initiative sets as a goal that cities should be able to distribute antibiotics to their entire population within 48 hours. However, it is uncertain how many large cities with a population similar to our model are prepared to meet this goal. Therefore, similar to [3], we consider a mass antiviral distribution timetable of 4 days. We assumed that an individual can thwart the disease 2 days on average after he starts receiving the drug. We also assume that the constant in the Harmonic number approximation is negligible. Therefore, $\varepsilon = ln\ (v)\ /(4+2)$. Considering $v = 10^7$, we have $\varepsilon = 2.6863$.

According to [3], drug treatment before the onset of rash offers 99% protection against mortality, and drug treatment 3 days post-onset of rash offers 80% protection against mortality. Thus, we estimate the preemption rate for susceptibles, incubators, and prodromals as $\tau = \varepsilon \times 0.99 = 2.6594$ (if the drug administering policy administers the drug to them), and the preemption rate for those in the early rash stage as $\mu = \varepsilon * 0.8 = 2.1490$. We assume that the drug is ineffective in the late rash stage.

All policies for administering drugs use $\mu = 2.1490$ as computed above. But $\tau$ will be 0 for some states in which the drug is not administered depending on the policy under consideration. Policy 1 administers drugs to anyone with fever or rash; thus, $\tau = 2.6594$ for prodromals. Policy 2 administers drugs only to patients with rashes; thus, $\tau = 0$ for prodromals. For both policies 1 and 2, $\tau = 0$ for susceptibles, early and late incubators. In policy 3, drugs are administered to everyone in a given cluster once the number of cases in the

cluster exceeds a certain threshold, otherwise only those with rash receive the drug. Thus, once the number of cases in a cluster exceed a certain threshold, $\tau = 2.6594$ for prodromals, susceptibles, early and late incubators, otherwise $\tau = 0$.

## B.3 Estimating the parameters pertaining to immunization

We now consider the additional parameters in the "vaccine only" scenario above and beyond those estimated in Appendix B.1. Refer to Table 8. According to [3], vaccines prevent disease with probability 1 (0.8, respectively) if administered to susceptibles (early incubators, respectively). According to [3, 7, 10], it would take approximately 10 days on average for cooperative individuals to get their shots in case of mass vaccination against an epidemic in a large U.S. city. The immunocompetent individuals are given the live vaccine (ACAM2000) which provides immediate immunity (if the vaccine is effective) [11]. Now, following the methodology in Appendix B.2, $\theta = \frac{ln(10^7)}{10} = 1.6118$ and $\pi = \frac{ln(10^7)*0.8}{10} = 1.2894$ (as in Appendix B.2, we consider that there are $v = 10^7$ individuals in the city under consideration). Immunodeficient individuals receive the Modified Vaccinia virus Ankara (MVA) vaccine [3, 6] that needs two-shots 30 days apart to provide immunity [11]. Thus $\tilde{\theta} = ln(10^7)/(10 + 30) = 0.4030$, and $\tilde{\pi} = ln(10^7) * 0.8/(10 + 30) = 0.3224$.

## B.4 Modifying the estimation for the vaccine and drug scenario

In this scenario, individuals can be preempted through either drug or vaccine. We estimate the parameters in Table 9. Vaccines are effective only when they are administered to individuals in susceptible or early incubation stage. Thus, preemption in later stages can only be through drugs. Referring to Table 9, $1/\tau$ and $1/\mu$ can therefore be directly obtained from the estimation strategy in Appendix B.2, i.e., $\tau = 2.6594$ and $\mu = 2.1490$.

The estimation of other parameters in Table 9 will depend on the policy for administering the drugs. Note that in policy 2, drugs are administered to only patients with rash. Thus, a susceptible or early incubator can be preempted only when he receives the vaccine. Thus $\Theta$, $\tilde{\Theta}$, $\Pi$ and $\tilde{\Pi}$ can be obtained directly from the respective values of $\theta, \tilde{\theta}, \pi, \tilde{\pi}$ estimated in Appendix B.3. Specifically $\Theta = 1.6118$, $\tilde{\Theta} = 0.4030$, $\Pi = 1.2894$ and $\tilde{\Pi} = 0.3224$. And, since the non-cooperatives do not receive vaccine, $\bar{\Theta} = \hat{\Theta} = \bar{\Pi} = \hat{\Pi} = 0$.

In policy 1 drug is administered to anyone with fever or rash. Thus, a susceptible or early incubator can be preempted only when he receives the vaccine, and $\Theta$, $\tilde{\Theta}$, $\Pi$, $\tilde{\Pi}$, $\bar{\Theta}$, $\hat{\Theta}$, $\bar{\Pi}$, and $\hat{\Pi}$ can be estimated identical

to the previous paragraph. The drug delivery parameter in the prodrome stage can be estimated as in the first paragraph, i.e., $\tau = 2.6594$.

In policy 3, drugs are administered to everyone in a given cluster once the number of cases in the cluster exceeds a certain threshold. Thus, in this case, susceptibles and early incubators may either be preempted through drugs or vaccines. We assume that any such immunocompetent individual is preempted through vaccination after an exponentially distributed wait time with parameter $\theta$, and is preempted through drug after an exponentially distributed wait time with parameter $\tau$. Thus, he is preempted after a minimum of the two wait times (if he receives the drug earlier than the vaccine he will be preempted through drug and vice versa). When the two exponential processes are independent, their minimum is an exponential process with parameter $\Theta$, where $\Theta = \theta + \tau$. Now from the estimations in Appendix A.4 and first 4 rows of Table 9, $\Theta = 1.6118 + 2.6594 = 4.2712$. For an immunodeficient individual the parameter for the vaccine is $\tilde{\theta} = 0.4030$ (from Appendix B.3). Thus, $\tilde{\Theta} = 0.4030 + 2.6594 = 3.0624$. Similarly, $\Pi = \pi + \tau = 1.2894 + 2.6594 = 3.9488$, and the $\tilde{\Pi} = \tilde{\pi} + \tau = 0.3224 + 2.6594 = 2.9818$. For late incubation, preemption can happen only through drug delivery, and the preemption rate as estimated in the first paragraph is $\tau = 2.6594$. The non-cooperative susceptibles and early incubators can only be preempted through drugs. Thus $\bar{\Theta} = 2.6594$, $\hat{\Theta} = 2.6594$, $\bar{\Pi} = 2.6594$, $\hat{\Pi} = 2.6594$.

## B.5 Estimation of the initial values of the state variables

First consider the “no countermeasure” scenario. The fraction of individuals who are in a given cluster and are initially infected (i.e., at $t = 0$) can be obtained from the number of initially infected individuals, total number of individuals overall and the distribution of the initially infected across clusters (Uniform, Central, Peripheral). Thus, at $t = 0$, the fraction of individuals who are in a given cluster and are susceptible can be calculated as the difference between the fraction of individuals in the cluster and the fraction of individuals who are in the cluster and are initially infected. We know that 20% of people in the United States are immunodeficient [12, 13, 14]. Thus, multiplying the above fraction with 0.2 (0.8, respectively) we obtain the fraction of individuals in a given cluster who are immunodeficient (immunocompetent, respectively) and susceptible.

We now consider the case that the initially infected individuals are all in prodrome stage. The fraction of individuals in a given cluster and in prodrome stage equals the fraction of individuals who are in the cluster and are initially infected. Next, multiplying the above fraction with 0.2 (0.8, respectively) we obtain the

fraction of individuals in a given cluster who are immunodeficient (immunocompetent, respectively) and in prodrome stage. All other state variables are 0 at $t = 0$.

When the initially infected individuals are distributed in various stages, the initial values of various state variables can be obtained similarly; only difference is that the fraction of individuals in the given stages of the disease at $t = 0$ are non-zero and can be obtained from the fraction of initially infected and the pattern of the distribution.

For the "drug only" scenario, the initial values of the state variables can be computed identically. Now, consider the scenarios in which vaccines are administered (that is, "vaccine only" and "both drug and vaccine" scenarios). Compared to the "drug only" scenario, each state variable (other than preempted and dead) is additionally characterized as cooperative and noncooperative. Consider the case that the initially cooperative individuals are uniformly distributed across clusters. First the initial values of each state variable corresponding to a cluster, a stage of the disease and immunodeficient/immunocompromised are calculated exactly as before. These values are subsequently multiplied with the initial cooperativity ($1-$ initial cooperativity, respectively) to give us the initial value of the corresponding state variable with the cooperative (noncooperative) qualifier. When the initially cooperative individuals are not uniformly distributed, then the only difference is that the initial cooperativity varies from cluster to cluster and can be obtained from the pattern of the distribution of the initially cooperative individuals, and for calculating the initial values of the state variables associated with a cluster the initial cooperativity for that cluster must be used. For example if the initially cooperative individuals are confined to one cluster, then initial cooperativity in all other clusters is 0. If all individuals in a cluster are initially cooperative, then initial cooperativity in the cluster is 1.

# References


1. Bailey M, Cao R, Kuchler T, Stroebel J, Wong A. Social connectedness: Measurement, determinants, and effects. Journal of Economic Perspectives. 2018 Aug;32(3):259-80.

2. Patacchini E. How does geographical distance affect social interactions? 2015 Jul. Available from: https://www.weforum.org/agenda/2015/07/how-does-geographical-distance-affect-social-interactions/

3. Finin P, Kosaraju A, Rose E, Rubin H. The role of vaccination, antiorthopoxvirus drug, and social cooperativity in a mathematical model of smallpox control. Biosecurity and bioterrorism: biodefense strategy, practice, and science. 2013 Mar 1;11(1):59-72.

4. Graeden E, Fielding R, Steinhouse KE, Rubin IN. Modeling the effect of herd immunity and contagiousness in mitigating a smallpox outbreak. Medical Decision Making. 2015 Jul;35(5):648-59.

5. Fenner F, Henderson DA, Arita I, Jezek Z, Ladnyi ID. Smallpox and its eradication. Geneva: World Health Organization; 1988 Mar 2.

6. Eichner M, Dietz K. Transmission potential of smallpox: estimates based on detailed data from an outbreak. American journal of epidemiology. 2003 Jul 15;158(2):110-7.

7. Kaplan EH, Craft DL, Wein LM. Emergency response to a smallpox attack: the case for mass vaccination. Proceedings of the National Academy of Sciences. 2002 Aug 6;99(16):10935-40.

8. Smith, R. Modelling disease ecology with mathematics. Springfield: American Institute of Mathematical Sciences; 2008.

9. Stroud C, Viswanathan K, Powell T, Bass RR, Committee on Prepositioned Medical Countermeasures for the Public. Current Dispensing Strategies for Medical Countermeasures for Anthrax. Prepositioning Antibiotics for Anthrax. 2011 Sep 30.

10. Halloran ME, Longini IM, Nizam A, Yang Y. Containing bioterrorist smallpox. Science. 2002 Nov 15;298(5597):1428-32.

11. Hatch GJ, Graham VA, Bewley KR, Tree JA, Dennis M, Taylor I, Funnell SGP, Bate SR, Steeds K, Tipton T, et al. Assessment of the protective effect of Imvamune and Acam2000 vaccines against aerosolized monkeypox virus in cynomolgus macaques. Journal of virology 87, 14 (2013), 7805–7815.

12. Macintyre CR, Costantino V, Chen X, Segelov E, Chughtai AA, Kelleher A, Kunasekaran M, Lane JM. Influence of population immunosuppression and past vaccination on smallpox reemergence. Emerging infectious diseases. 2018 Apr; 24(4):646.

13. Bavarian Nordic delivers 1 million doses of first vaccine developed under U.S. biopreparedness program to the Strategic National Stockpile. 2010 [cited 21 May 2020]. Available from: http://www.bavarian-nordic.com/investor/news/news.aspx?news=2041

14. Kemper AR, Davis MM, Freed GL. Expected adverse events in a mass smallpox vaccination campaign. Effective Clinical Practice. 2002 Mar;5(2).
