# Supplementary material for: Countering the potential re-emergence of a deadly infectious disease—Information warfare, identifying strategic threats, launching countermeasures
Source: PLoS One. 2021 Aug 20;16(8):e0256014. doi: 10.1371/journal.pone.0256014 (PMC8378755; doi:10.1371/journal.pone.0256014)
Supplement: Attachment — Submitted filename: Letter to Academic Editor.pdf [file pone.0256014.s005.pdf]

The Academic Editor has made two concrete suggestions based on the reviews of the revision we had submitted in the previous round. We have complied with the suggestions and provide the details pertaining to our compliance next.

RESPONSE TO THE COMMENTS OF THE ACADEMIC EDITOR

==============================

As you can see the reviewers positively evaluated your manuscript. However, both of them remarked a difficulty for the readability of the manuscript due to its length. I suggest to slightly modify the results section, adding a small summary of the main finding at the end of each subsection. Moreover, it would be better to number the section and the subsections.

==============================

We have complied with both the suggestions of the Academic Editor. Specifically, we have added a paragraph at the end of each subsection of the Result Section in which we have summarized the main finding in the subsection. Please refer to the text in red on p. 21 (Section 3.1), p. 25 (Section 3.2), pp. 30, 31 (Section 3.3), p.36 (Section 3.5). There was already such a paragraph at the end of Section 3.4 in the previous submission (p. 31 in the previous submission) which we have retained in the version we are submitting (pp. 31, 32 in the current version). We have also numbered the sections and subsections as advised by the Academic Editor. Now that we have numbered the sections and subsections, we have utilized the numberings to refer to the content as necessary in the rest of the paper (text in red on pp. 7, 10, 15, 30, 36 - 39, 52, 58). We hope that both these modifications enhance the readability of our work and we thank the Academic Editor for the thoughtful suggestion.

Journal Requirements:

Please review your reference list to ensure that it is complete and correct. If you have cited papers that have been retracted, please include the rationale for doing so in the manuscript text, or remove these references and replace them with relevant current references. Any changes to the reference list should be mentioned in the rebuttal letter that accompanies your revised manuscript. If you need to cite a retracted article, indicate the article's retracted status in the References list and also include a citation and full reference for the retraction notice.

We confirm that we have reviewed our reference list and that it is complete and correct. We did not cite any paper that has been retracted.
