# Supplementary material for: Countering the potential re-emergence of a deadly infectious disease—Information warfare, identifying strategic threats, launching countermeasures
Source: PLoS One. 2021 Aug 20;16(8):e0256014. doi: 10.1371/journal.pone.0256014 (PMC8378755; doi:10.1371/journal.pone.0256014)
Supplement: Attachment — Submitted filename: renamed_e1593.docx [file pone.0256014.s003.docx]

**COMMENTS – Manuscript PONE-D-20-32001**

Title: “Countering the potential re-emergence of a deadly infectious disease - information warfare, identifying strategic threats, launching countermeasures”

**Important note: This review pertains only to ‘statistical aspects’ of the study and so** **‘****clinical aspects’ [like medical importance,** **relevance of the study,** **‘clinical significance and** **implication(s)’ of the whole study, etc.] are to be evaluated [should be assessed] separately/independently. Further please note that any ‘statistical review’ is generally done under the assumption that (such) study specific methodological [as well as execution] issues are perfectly taken care of by the investigator(s). This review is not an exception to that and so does not cover clinical aspects {however, seldom comments are made only if those issues are intimately / scientifically related & intermingle with ‘statistical aspects’ of the study}. Agreed that ‘statistical methods’ are used as just tools here, however, they are vital part of methodology [and so should be given due importance].**

**COMMENTS:** Your ABSTRACT is well drafted but assay type. Please note that it is preferable [though this article is not of ‘usual’ type] to divide the ABSTRACT with small sections like ‘Objective(s)’, ‘Methods’, ‘Results’, ‘Conclusions’, etc. [refer to item 1b of CONSORT checklist 2010: Structured summary of trial design, methods, results, and conclusions] which is an accepted practice of most good/standard journals [including PLOS-ONE]. It will definitely be more informative then, I guess, whatever the article type may be {even if section headings may be different considering nature of this article}.

Surprisingly, in ‘Abstract’ the complete account [though very nice] is focused on ‘smallpox’ and no mention of ‘COVID-19’ {agreed, the article is on ‘Countering the potential re-emergence of a deadly infectious disease’ some mention of current pandemic / COVID-19 outbreak was/is expected to emphasize present context / implications / applications (mainly because at the end of ‘Introduction’ section, it is said that “we expect that our model would easily port to other infectious diseases such as COVID-19 through the consideration of a different set of disease states and parameters.”}. Many readers (as you are aware) decide to read full text on the basis of ‘abstract’.

Overall, the article is nicely presented, perfect by all the means, 100% correct/accurate (scientifically). However, there are few small questions: Why the table headings are below the tables? (usual practice is to place them above). Was it necessary to cover all possible scenarios [(1) No countermeasure - neither drugs nor vaccines administered, (2) Vaccines only, (3) Drugs only, (4) Both drugs and vaccines.] in such details? Could not avoid/reduce number of figures? (there are 17 which is too much, I guess). Is it essential to include such a lengthy ‘appendix’ {A. Equations of Smallpox disease dynamics, B. Parameter estimation i.e. pages 33 to 49}?

Finally, I have a fundamental/basic question (though the entire article/study is faultless) is this a right place for such an article? I humbly request the ‘Editor/Chief editor’ to kindly think over.
